# Research progress on the physiological response and molecular mechanism of cold response in plants

**DOI:** 10.3389/fpls.2024.1334913

**Published:** 2024-01-30

**Authors:** Yong Wang, Jin Wang, Rehman Sarwar, Wei Zhang, Rui Geng, Ke-Ming Zhu, Xiao-Li Tan

**Affiliations:** School of Life Sciences, Jiangsu University, Zhenjiang, China

**Keywords:** low-temperature stress, physiological and biochemical metabolism, signal transduction, hormones, cold resistance mechanism

## Abstract

Low temperature is a critical environmental stress factor that restricts crop growth and geographical distribution, significantly impacting crop quality and yield. When plants are exposed to low temperatures, a series of changes occur in their external morphology and internal physiological and biochemical metabolism. This article comprehensively reviews the alterations and regulatory mechanisms of physiological and biochemical indices, such as membrane system stability, redox system, fatty acid content, photosynthesis, and osmoregulatory substances, in response to low-temperature stress in plants. Furthermore, we summarize recent research on signal transduction and regulatory pathways, phytohormones, epigenetic modifications, and other molecular mechanisms mediating the response to low temperatures in higher plants. In addition, we outline cultivation practices to improve plant cold resistance and highlight the cold-related genes used in molecular breeding. Last, we discuss future research directions, potential application prospects of plant cold resistance breeding, and recent significant breakthroughs in the research and application of cold resistance mechanisms.

## 1 Introduction

The plant production process faces various adverse conditions, including salt-alkali, high temperature, low temperature, drought, pests, and other stresses. One of the common challenges is low temperature stress, which can be classified as cold damage (>0°C) and freezing damage (<0°C) (Ding et al., 2019). Both cold damage and freezing damage have detrimental effects on crop yield and quality (Aslam et al., 2022). They inhibit plant respiration and metabolism, reduce root water absorption rates, and lead to the yellowing of leaves and browning of fruits. Prolonged exposure to temperatures below 0°C damages the cell membrane, resulting in cell death. Even when the temperature is above 0°C and no ice is present, water balance and physiological activities can still be disrupted, causing injury and death of plants.

Plants in the growth stage are particularly sensitive to temperature, and low temperatures significantly reduce flowering and fruit yield in tomatoes (Yang et al., 2021; Iovane and Aronne, 2022). Studies have shown that *Capsicum annuum* is susceptible to cold damage during postharvest storage and transportation, leading to symptoms such as concave skin surface, concave spots, brown calyx, water, and nutrient loss (Ding and Wang, 2018; Kong et al., 2018). In the case of *Solanum tuberosum*, different low-temperature treatments have varying effects. Leaves of *S. tuberosum* above 0°C can maintain normal growth, but temperatures below −2°C cause serious damage, such as wilting, leaf drop, waterlogging, and even death (Yan et al., 2021). Low-temperature stress inhibits seed germination and increases seedling mortality in *Brassica napus* (Zhu et al., 2021). *Cucumis sativus*, a cold-sensitive plant, exhibits reduced germination rates, shriveled and yellowish leaves, decreased seed set rates, and increased susceptibility to rot during storage and transport after cold stress (Zhang et al., 2021). *Solanum melongena* leaves undergo elongation, become soft and atrophied, and curl completely under low-temperature stress, with light green leaf spots appearing. *Zea mays* growth is adversely affected by cold stress, resulting in inhibited germination, stunted grain development, and a significant decrease in yield (Peleg and Blumwald, 2011).

However, most plants have evolved a strategy known as cold acclimation to adapt to low-temperature environments. This strategy enhances their cold tolerance by triggering physiological changes and cold response gene expression through a cascade of molecular events (Browse and Xin, 2001). Currently, the molecular mechanism underlying plant resistance to low temperatures remains insufficiently understood. Therefore, there is an urgent need to analyze the regulatory mechanism of plants under low-temperature stress and develop technology to improve the plants’ resistance. This article comprehensively reviews the molecular mechanisms involved in plant responses to low-temperature stress, and the advances made in understanding the physiological, biochemical, and molecular signaling mechanisms underlying plant cold resistance. The roles of transcription factors (TFs), including C repeat binding factor (CBF), CBF expression inducer 1 (ICE1), APETALA2/ethylene-responsive factor (AP2/ERF), and dehydration-responsive element binding (DREB1), are extensively discussed in response to cold stress. It provides a theoretical basis for high-quality, high-yield, and stress-resistant plant cultivation and practical guidance for plant cold tolerance research.

## Signaling pathway of plants’ response to low-temperature stress

### 2.1 Low-temperature perception and transduction of calcium signals

**Figure 1:**
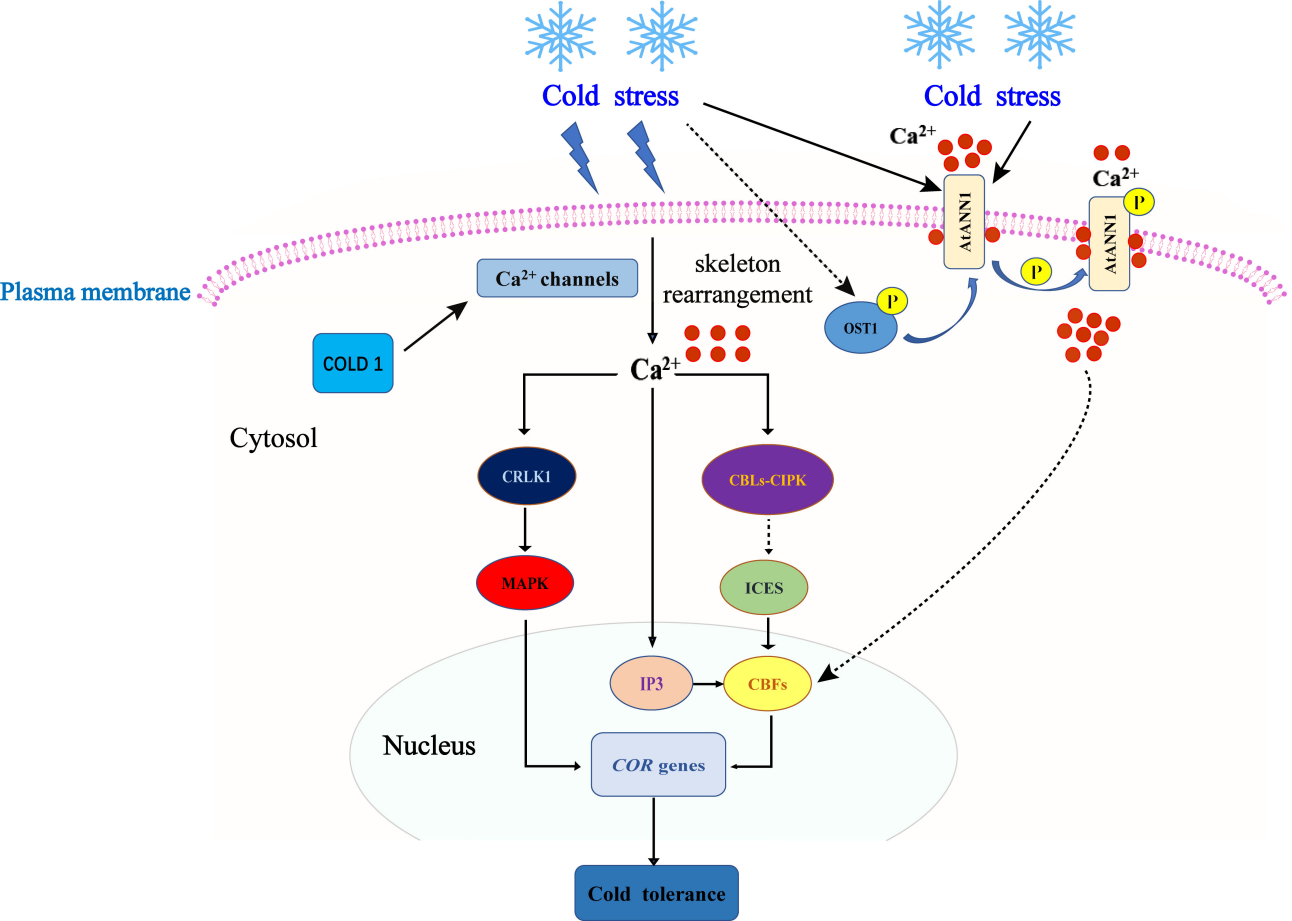
Ca^2+^ signal perception and transduction models. Cold stress induces extracellular Ca^2+^ influx, triggering the Ca^2+^ signal. Ca^2+^ promotes the production of IP3, further enhances the expression of the *COR* gene, and enhances plant cold tolerance. In *Arabidopsis thaliana*, OST1 kinase can regulate the expression of *CBF* and *COR* genes through phosphorylation of AtANN1 to enhance cold tolerance. Low temperature can activate the MAPK signaling pathway to regulate the expression of the downstream *COR* gene and enhance cold tolerance.

Calcium (Ca^2+^) serves as a universal second messenger in plant cells and plays a crucial role in plant growth, development, and responses to various biotic and abiotic stresses, such as cold, heat, and salt stress (Jiang et al., 2019). When exposed to low temperatures, changes in membrane fluidity and cytoskeletal rearrangement occur, leading to an influx of Ca^2+^ and an increase in intracellular Ca^2+^ levels (Dodd et al., 2010). The increased intracellular flow of Ca^2+^ is sensed by downstream calcium signal receptors and transmits Ca^2+^ signals further downstream, thus participating in signal transduction to induce the expression of cold response genes (Sanyal et al., 2016). Several calcium-responsive proteins, including calmodulin (CaM), CAM-like proteins (CML), calcium-dependent protein kinases (CDPK), and calcineurin B-like proteins (CBL), have been identified and play significant roles in responding to low-temperature stress (Reddy et al., 2011; Ma et al., 2015; Zhu, 2016). Studies conducted in *A. thaliana* have shown that the level of gene transcription induced by low temperature is positively correlated with the intensity of Ca^2+^ signals (Kleist and Luan, 2016). An increased concentration of intracellular Ca^2+^ promotes the production of inositol triphosphate (IP3), which amplifies the Ca^2+^ signal by regulating Ca^2+^ channels and further enhances the expression of cold response genes such as *CBF* and *COR* genes (Sulaiman et al., 2012). The specific components involved in the regulation of calcium signaling have been elucidated. For instance, the Ca^2+^ transporter *AtANN1* contributes to cold-induced calcium signaling by positively regulating cold tolerance through its effects on Ca^2+^ influx and the downstream CBF-COR-dependent cold signal transduction pathway in *A. thaliana* (Liu et al., 2021c). Similarly, the low-temperature receptor protein COLD1 interacts with G proteins to activate Ca^2+^ channels and enhance cold tolerance in *O. sativa* (Nawaz et al., 2014). Furthermore, calcineurin B-like protein-interacting protein kinase (CIPK) is another crucial component of Ca^2+^ signaling in the stress response. CIPK specifically interacts with CBLs, which control the localization and activation of CIPK. The CBL-CIPK signaling pathway plays a critical role in Ca^2+^ signaling in plants and is involved in the response to cold stress (Ormancey et al., 2017). Furthermore, a study (Yang et al., 2010) demonstrated that CRLK1 is a positive regulator of the plant response to low-temperature stress through gene knockout and complementation experiments. CRLK1 interacts with MEKK1 to activate the MAPK cascade reaction. The mitogen-activated protein kinase (MAPK) cascade has been extensively studied for its role in the response to low-temperature stress. This cascade typically consists of three protein kinases: MAP kinases (MAPKKK, MAP3K, or MEKK), MAP kinases (MAP2K, MKK, or MEK), and MAP kinases (MAPK). MKK2 and MAPKKK kinase MEKK1 are simultaneously activated by cold stress, and MKK2 induces the expression of *COR* genes, thereby improving plant cold tolerance (Teige et al., 2004). The MAP2K/MKK2 signaling pathway is activated by low temperatures and regulates the expression of downstream *COR* genes (Teige et al., 2004). While significant progress has been made in understanding the Ca^2+^ regulation mechanism in the plant response to cold stress, further research is needed to explore the interaction between Ca^2+^-responsive transcription factors and the Ca^2+^ signaling pathway (**Figure 1**).

### 2.2 Signaling pathway and regulatory mechanism in response to low-temperature stress

#### 2.2.1 CBF-dependent low-temperature response signaling pathway

**Figure 2:**
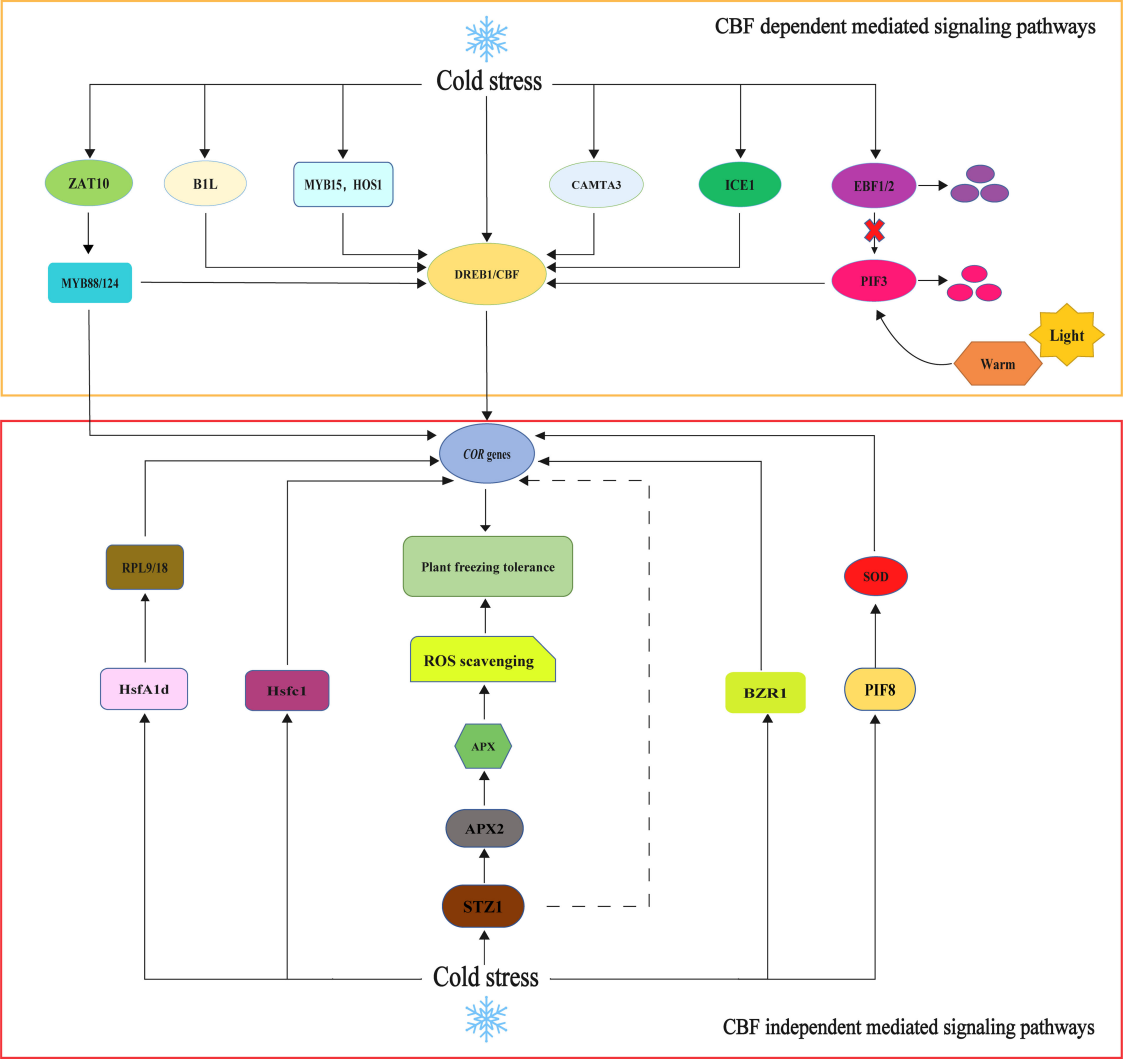
CBF-dependent and CBF-independent mediated signaling pathways models. Among the CBF-dependent pathways. The CBF-COR pathway is the primary cold stress signal regulation pathway. Cold stress can activate BIL, MYB15, HOS1, CAMTA3, ICE1, and ZAT10; regulate the expression of the *CBF* gene, thereby enhancing the expression of the *COR* gene; and regulate plant cold resistance. PIF3 can bind the promoter region of *CBFs*, inhibit the expression of *CBFs* and its downstream cold response genes, and negatively regulate the cold resistance of *Arabidopsis thaliana*. Under normal temperature and light conditions, EBF1/2 targets degrade PIF3 and induce *CBF* gene expression. Low-temperature and dark conditions promote the degradation of EBF protein, which makes PIF3 protein more stable. In the CBF-independent pathway, HsfA1d, Hsfc1, STZ1, BZR1, PIF8, and other transcription factors can directly regulate the expression of the *COR* gene independently of CBF and regulate plant cold tolerance.

The CBF-dependent low-temperature response signaling pathway is an essential mechanism for plants to cope with cold stress. CBF, which stands for C-repeat binding factor, is a group of plant-specific transcription factors that play a crucial role in regulating the expression of COR (cold-regulated) genes (Stockinger et al., 1997). The signaling pathway of *CBF* in response to low-temperature stress in higher plants has been extensively investigated. In the model plant *A. thaliana*, *CBF* genes belong to the dehydration-responsive element-binding protein (DREB) subfamily. They are regulated by the upstream transcription factor ICE (inducer of *CBF* expression) (Li et al., 2020). Consequently, *CBFs* are also known as *DREB1* genes, and their activation induces the downstream expression of *COR* genes to improve plant cold tolerance. This ICE-CBF-COR cascade is crucial in maintaining normal growth and development during cold stress. Several transcription factors, including the positive regulator CAMTA (calmodulin binding activator of transcription) and negative regulators such as MYB15 and HOS1, directly regulate *CBF* gene expression (Ding et al., 2019). When plants are exposed to cold stress, the signal transduction process is triggered, leading to the activation of ICE. ICE, in turn, promotes the transcription and expression of *CBF1/2/3* genes, which enhances the expression of *COR* genes (Hwarari et al., 2022). The ICE-CBF protein is regulated by ubiquitin, which improves plant cold tolerance (Sharma et al., 2021). Research has shown that the *ICE1* transcription factor is crucial for the expression of DREB1/CBF (Thomashow and Torii, 2020). ICE1, a structural protein of the basic helix–loop–helix (bHLH) domain of the MYC transcription factor, specifically recognizes cis-acting elements of MYC (CANNTG) in the promoter region of *CBF3/DREB1A* and binds to them, inducing the expression of *CBF/DREB1* and activating the cold tolerance regulatory pathway (Lee et al., 2005a; Wang et al., 2008). A study of *A. thaliana* discovered that the *ice1* mutation hampers *CBF1* expression, leading to decreased downstream expression of the *CBF* gene, while overexpression of ICE1 effectively enhances *CBF* expression and improves cold tolerance in *A. thaliana* (Chinnusamy et al., 2003). Furthermore, PUB25 and PUB26 interact with ICE1 to ubiquitinate ICE1 and stabilize its protein levels. The stabilized ICE1 protein then interacts with MYB15 to inhibit its DNA-binding activity, ultimately activating *CBF* gene expression (Wang et al., 2023c). Furthermore, MYB15, ICE1, and HOS1 have been identified as direct regulators of *CBF/DREB1* expression. ICE1 can interact with MYB15 and directly bind to the *MYB15* promoter region, leading to the downregulation of *MYB15* expression under cold stress (Agarwal et al., 2006b). The interaction between B1L (BYPASS1-LIKE) and TTL (TRANSTHYRETIN-LIKE) participates in the regulation of plant growth, development, and cold tolerance (Chen et al., 2020). The 14-3-3 proteins are a highly conserved family of acidic proteins widely expressed in eukaryotes and involved in various biological processes. B1L improves the stability of *CBF* by interacting with 14-3-3λ, thus inducing the expression of *COR* genes and improving cold tolerance in *A. thaliana* (Chen et al., 2019). ABI4 is a transcription factor of AP2/ERF (APETALA2/ethylene responsive factor). ABI4 can regulate downstream gene expression by recognizing promoter motifs (Chandrasekaran et al., 2020). MdABI4 interacts with MdICE1 to enhance the transcriptional regulation of the downstream target gene *MdCBF1* by *MdICE1*. MdABI4 integrates jasmonic acid and abscisic acid signals to positively regulate cold tolerance in *M. domestica* through the JAZ-ABI4-ICE1-CBF cascade (An et al., 2022). PIF3 (phytochrome-interacting factors 3) is a bHLH transcription factor and a member of the PIF protein family (Ni et al., 1998). PIF3 acts as a negative regulator of plant cold tolerance by inhibiting the expression of the *CBF* gene. Two F-box proteins, EBF1 (EIN3-BINDING F-BOX 1) and EBF2 (EIN3-BINDING F-BOX 2), directly target PIF3 for degradation through the 26S proteasome pathway. This degradation leads to the activation of *CBF* gene expression (Jiang et al., 2017). Trx-h2 (Thioredoxin h2) interacts with CBFs at low temperatures to regulate CBF activity and protein structure. This interaction activates downstream *COR* gene expression and improves plant cold tolerance (Lee et al., 2021). Dehydrin, also known as LEA D-11 or LEA II (late embryogenesis abundant) protein induces cold acclimation in freezing-tolerant plants, enabling plants to survive under adverse conditions (Kosová et al., 2007). Dehydrin accumulates in plants when they are exposed to cold. Vernalization, the prolonged exposure of plants to low temperatures, is associated with a significant reduction in dehydrin accumulation, which delays the acquired frost tolerance of the plant (Kosová et al., 2011). There is a positive correlation between the relative accumulation of dehydrin protein and winter survival (WS) in winter wheat and barley (Vítámvás et al., 2019). Cold-induced dehydrin Lti30 binds to the membrane through its conserved K segment and reduces the membrane phase transition temperature (Eriksson et al., 2016). Lti30 binds to the lipid bilayer electrostatically, limiting the mobility of lipid and bound protein molecules and protecting the cell membrane by forming aggregates (Gupta et al., 2019). As a cold-induced CBF transcription factor in the *A. thaliana* genome, CBF1/2/3 can bind to the CRT/DRE/LTRE elements, thus increasing the tolerance to plant cold (Gilmour et al., 2004). Several dehydrin genes were identified in cold regulatory proteins whose promoters contained elements of CRT/DRE/LTRE (Zolotarov and Strömvik, 2015). Under cold stress, the ICE1-CBF cold response signaling pathway gene of the *DIICE1* overexpression strain was significantly upregulated compared with the wild type (Yang et al., 2019). In conclusion, the CBF-dependent low-temperature response signaling pathway involves multiple transcription factors and protein interactions that collectively regulate the expression of COR genes and enhance plant cold tolerance (**Figure 2**).

#### 2.2.2 CBF-independent signaling pathways for low-temperature response

The regulation of *COR* genes is crucial for enhancing plant tolerance to cold during cold acclimatization. However, recent studies have revealed that only 10%–20% of *COR* genes in plant cells are regulated by CBF, while the majority of them participate in cold tolerance signaling pathways independently of CBF (Ding et al., 2019). For instance, the overexpression of *HsfC1* (heat shock transcription factor C1) in *A. thaliana* induces the expression of *COR* genes and improves cold tolerance (Jiao et al., 2022). A genome-wide association study on hypocotyl growth in *A. thaliana* demonstrated that *HsfA1d* (heat shock transcription factor A1d) promotes hypocotyl elongation under cold stress by binding to the promoters of two ribosomal protein genes, *RPL9* and *RPL18*, thus enhancing plant tolerance to low temperatures (Liu et al., 2021a). Moreover, the transcriptional regulatory factor BZR1 in the brassinosteroid (BR) signaling pathway regulates the expression of *CBF1* and *CBF2* and also independently controls *COR* genes. BZR1 regulates plant cold tolerance through both CBF-dependent and CBF-independent pathways (Li et al., 2017b). Initially identified as a *CBF-independent* gene in the cold tolerance signaling pathway, ZAT10 is downstream of *MAPK*, *APX*, and *CAT* (Gong et al., 2020). ZAT10 induces the expression of *COR* genes by regulating the expression of *MYB88/124*, thus enhancing the cold tolerance of *M. domestica* (Li et al., 2023). The overexpression of *CsPIF8* in *S. lycopersicum* and *V. vinifera* callus enhances citrus cold tolerance by increasing SOD activity and reducing O_2_ levels, indicating that *CsPIF8* is involved in regulating cold tolerance independently of the CBF pathway (He et al., 2020). PeSTZ1 is induced under low temperature stress and directly binds to the PeAPX2 promoter, promoting its activation and reducing ROS accumulation under cold stress. This regulation of gene expression improves the cold resistance of *Populus euphratica* by regulating the expression of *COR* genes (He et al., 2019). Research has shown that BnHY5 regulates hypocotyl length in *B. napus* by regulating the downstream gene *PIF4* in the IAA pathway. It is speculated that *BnHY5* is a key gene involved in cold resistance and hypocotyl development in *B. napus* (Jin et al., 2022). Numerous CBF-independent signaling pathways for low-temperature response exist in plants, and further exploration is required to elucidate their regulatory mechanisms (**Figure 2**).

## 3 Low-temperature effect on the physiological and biochemical metabolism of plants

### 3.1 Effect of low temperature on membrane stability

The cell membrane, primarily composed of phospholipids, is highly sensitive to temperature and plays a crucial role in responding to low-temperature stimuli. It acts as the main sensor for abiotic stress signals, allowing plant cells to detect changes in cell membrane fluidity and protein conformation caused by low-temperature stress. Cell membrane fluidity, regulated by lipid composition and distribution, is a key factor in plant adaptation to temperature changes (Li et al., 2016). Prolonged exposure to cold temperatures can alter the lipid composition of plant cell membranes, causing a transition from a flexible, liquid crystal phase to a more rigid, solid gel phase. This decrease in membrane fluidity can impair its functionality and disrupt intracellular metabolism. Additionally, membrane and membrane-binding enzyme activities may be affected (Sanchez et al., 2019). During freezing stress, when the temperature drops below 0°C, ice crystals can form in plant tissues, potentially leading to plant death. The obstruction of the cell membrane prevents intracellular fluid from freezing while extracellular water molecules form ice crystals. This rapid increase in osmotic pressure of the extracellular fluid can damage the cell membrane and denature its proteins, ultimately resulting in the loss of cellular integrity (Wang et al., 2023d). Low-temperature stress induced by freezing or cold temperatures also directly affects membrane integrity, molecular transport, cell signaling, and respiratory functions during seed germination (Hassan et al., 2021). Exposure to cold stress can increase membrane permeability, causing the leakage of essential electrolytes and cellular substances, such as amino acids, carbohydrates, unsaturated fatty acids, and metabolic compounds. This disturbance in ion balance inside and outside the cell can cause cell damage or death. Studies have shown that a higher unsaturated fatty acid content in the membrane of *A. hypogaea* corresponds to a lower membrane lipid phase transition temperature and stronger cold resistance (Sui et al., 2018). *Arabidopsis thaliana* exhibited a significant reduction in membrane lipid composition and severe membrane structure damage under cold stress (Kenchanmane Raju et al., 2018). Membrane stability is crucial for the survival of plant seedlings under low-temperature stress, and cold acclimation is closely associated with dynamic changes in lipid composition (Zheng et al., 2011; Degenkolbe et al., 2012). Exposing plants to low-temperature conditions can lead to bursts of ROS, which can induce membrane lipid peroxidation and increase levels of malondialdehyde (MDA). MDA can react with proteins and nucleic acids, disabling their function (Morales and Munné-Bosch, 2019). This process significantly increases membrane permeability, causing electrolyte leakage and irreparable cell damage. Therefore, MDA levels are used as an evaluation marker for lipid peroxidation, reflecting the extent of cell membrane damage and plant stress resistance (Morales and Munné-Bosch, 2019).

### 3.2 Influence of low temperature on fatty acid content

Lipids are essential components of biomembranes and play crucial roles in signal transduction and stress response (Suh et al., 2022). Moreover, they serve as fundamental building blocks for cell membranes, cuticle proteins, and wax constituents, forming a structural barrier against environmental stress (Upchurch, 2008). The activity of alpha-linolenic acid lipase contributes to the restoration of membrane fluidity (Grechkin, 1998), potentially regulating plant defense gene expression against cold stress and enhancing plant resilience (Kachroo et al., 2001). Fatty acids and their derivatives play crucial roles in various biological processes, including growth, development, and cellular metabolism in plants and related tissues (Lee et al., 2003). During the reproductive development of male flowers, fatty acids play a vital role in forming the anther cuticle and pollen wall, ensuring fertility (Wan et al., 2020). The plant cell membrane, consisting of a phospholipid bilayer in a liquid crystal phase, undergoes a temperature-dependent transformation of glycerol ester, closely related to the degree of fatty acid unsaturation. Cold-resistant plants have higher levels of unsaturated membrane lipids, resulting in a lower transition temperature compared to cold-sensitive plants(Lyons, 1973). Low temperatures significantly reduce membrane lipid fluidity and contraction, immobilizing membrane proteins and altering the controlled entry/exit of substances, which can induce plant metabolic disorders (Sevillano et al., 2009). The fluidity and stability of the cell membrane are closely associated with plant cold resistance. Generally, plants with greater membrane fluidity and stability exhibit higher cold tolerance (Wu et al., 2022). The presence of unsaturated fatty acids helps maintain the fluidity of the cell membrane in plants (Miquel et al., 1993; Upchurch, 2008). Plants primarily regulate fatty acid desaturase gene activity to influence the composition of membrane fatty acids. Lipid desaturation is a common stress response aimed at maintaining membrane fluidity under low temperature stress (Kenchanmane Raju et al., 2018). Correlation studies have shown that cold-tolerant varieties of *Medicago sativa* have higher total and unsaturated fatty acid contents than sensitive varieties under low temperature stress (Tian et al., 2022). Furthermore, *S. tuberosum* acclimated to cold storage stores linoleic acid in leaf tissue membrane glycerides (18:2) during cold stress (Vega et al., 2004). Hexadecanetrienoic acid (16:3) and linolenic acid (18:3) are important components of membrane lipids. Increasing the TA content in chloroplast membranes enhances plant tolerance to cold during early growth stages (Iba, 2002). Moreover, low-temperature cold stress treatment significantly increases galactosyl diglyceride (DGDG) content in cotyledon lipids of *Phaseolus vulgaris* varieties, enhancing cold resistance (Wang et al., 2020b). Lipidomic studies of *Sorghum bicolor* after cold stress revealed increased conservative and cooling-induced lipid unsaturation, increased phosphatidylcholine unsaturation during cooling, and higher galactoid unsaturation (Marla et al., 2017). Investigations into the effect of low temperature on fatty acid unsaturation in *Lagenaria siceraria* and *cucumber* roots measured membrane fluidity using the double bond index (DBI) and showed that excessive accumulation of linolenic acid may trigger a hyperbolic reaction. Additionally, cold stress upregulates lipoxygenase (LOX) activity in *Ficus carica* leaves and gourd roots. The level of unsaturated lipids in the plasma membranes of the root is closely associated with a plant’s cold tolerance capacity (Lee et al., 2005b). Linolenic acid serves as a precursor for synthesizing JA, which is formed after oxidation. Methyl jasmonate (MeJA) reduces oxidation and damage to plants during storage at low temperatures, thus promoting economic benefits (González-Aguilar et al., 2000; Ding et al., 2002; Gong et al., 2022). MeJA affects the expression of genes related to cold tolerance, protecting plants from low-temperature stress (Laura et al., 2018).

### 3.3 Influence of low temperature on organic osmolytes

Plants use two main mechanisms to accumulate organic osmolytes: uptake of inorganic ions from the external environment and synthesis of organic solutes within the cell. Osmotic regulation is crucial for plant protection against stress and relies on intracellular biosynthesis and absorption of specific substances. During abiotic stress responses, active solute accumulation and increased concentration of cell-soluble apoplastic fluid help maintain osmotic pressure, prevent excessive water loss, and protect against cell protoplast death, thereby enhancing plant stress resistance. The accumulation of soluble sugars (such as glucose, sucrose, fructose, and galactose) and soluble proteins is positively associated with plant cold resistance. These compounds help maintain osmotic potential, enhance water retention capacity, reduce the freezing point of cell fluids, alleviate damage to membrane structures under low-temperature stress, and prevent plant death. Free proline plays a role in maintaining the osmotic pressure balance between protoplasts and the surrounding environment. It promotes protein hydration after low-temperature stress, protects the spatial structure of enzymes, and improves plant resistance to low temperatures, thereby reducing damage (Fedotova and Dmitrieva, 2016). Proline promotes protein structural stability and membrane integrity through hydrogen bond interactions (Trovato et al., 2019). Studies have demonstrated that the contents of soluble sugars, proteins, and proline correlate with cold tolerance in plants (Li et al., 2012). *A. hypogaea* exhibits increased accumulation of organic osmolytes under cold stress, and cultivars with strong resistance to cold can accumulate higher levels of organic osmolytes to resist stress (Kavi Kishor and Sreenivasulu, 2014). Measuring proline and soluble sugar concentrations in *O. sativa* plants overexpressing *OsLTPL159^IL112^* after low temperature treatment showed that this overexpression improved the cold tolerance of early *O. sativa* seedlings by promoting cellulose deposition and enhanced the accumulation of organic osmolytes (Zhao et al., 2020). On the other hand, disruption of the proline-rich protein encoded by *OsPRP1* in *O. sativa* increased the plant’s sensitivity to low temperatures at the seedling stage (Nawaz et al., 2019). Proline levels are positively associated with plant cold tolerance and can indicate cold tolerance and low-temperature stress (Ghosh et al., 2022). Long-term low-temperature stress increased the osmotic pressure of maize leaves and reduced cell damage (Guo et al., 2022). The content of betaine can also serve as an index to evaluate the plant’s tolerance to cold. Betaine improves resistance to low temperature in plants under osmotic stress, maintains the stability of the structure and function of biological macromolecules (Huang et al., 2000), promotes seed germination (Park et al., 2004), enhances plant antioxidant capacity (Wang et al., 2021a), and plays a vital role in maintaining normal respiration and photosynthesis.

### 3.4 Influence of low temperature on photosynthesis

Photosynthesis, the fundamental process for crop and energy plant production, is highly sensitive to changes in environmental temperature (Chen et al., 2015). Research has shown that 90%–95% of a plant’s dry weight is derived from photosynthesis products, making them highly vulnerable to low-temperature stress (Chassot et al., 2001; Bilska and Sowinski, 2010). Many aspects of photosynthesis are affected by low temperature, including chloroplast structure, transpiration rate (Tr), photosynthetic pigment content, net photosynthetic efficiency (A), stomatal conductance (Gs), photochemical reactions, and the transport and distribution of photosynthetic products (Zhang et al., 2022a). For example, low-temperature treatment reduces the dry matter content of *A. hypogaea* seedlings, leads to a decrease in Gs and the transpiration rate of leaves, and causes a decrease in the photosynthetic rate (Pn) due to the interplay between stomatal and nonstomatal factors (Liu et al., 2013). Chloroplasts, which are one of the earliest organelles to sense low temperatures, play a vital role in plant growth and development (Liu et al., 2018). Low temperatures result in the destruction of organelle ultrastructure and affect the concentration of metabolites in chloroplasts (Ambavaram et al., 2014). Exposure to cold stress alters chloroplast membrane states and enzyme activities, leading to a decrease in photosynthetic effectiveness and the accumulation of excessive reactive oxygen species (ROS) and aberrant thylakoid morphology. Enzymatic reactions may slow down, and the high levels of ROS can damage membrane structures (Gan et al., 2019). These factors can lead to chloroplast dysfunction and degradation, ultimately affecting the activity of the light system (Liu et al., 2018). Under low-temperature stress, C_3_ plants, such as chrysanthemum, exhibit reduced maximum PSII photochemical efficiency (*Fv/Fm*), PSII quantum yield, light energy capture efficiency, and apparent quantum efficiency, which inhibits photosynthesis (Ozturk et al., 2013). Wheat leaves subjected to low temperatures also experience a decrease in the photosynthetic rate, stomatal conductance, and transpiration rate, which impacts wheat’s photosynthetic activity and leads to decreased yield (Zhang et al., 2022a). Low temperature diminishes the transfer of light energy and the conversion efficiency of PSII on the thylakoid membrane, reducing the capacity to absorb CO_2_ and the electron transport ability, ultimately affecting photosynthesis and the formation and transport of photosynthetic products (Paredes and Quiles, 2015). Additionally, following low-temperature treatment, genes related to photosynthetic pathways are downregulated in *B. napus*, inhibiting the photosynthetic system, reducing the maximum quantum efficiency of *Fv*/*Fm*, and affecting light response and the Calvin cycle (Hussain et al., 2022). In conclusion, low temperatures can impact the efficiency of light energy use in plants, leading to a decrease in enzyme activity and ultimately affecting plant photosynthesis.

### 3.5 Influence of low temperature on oxidative stress

Reactive oxygen species (ROS) are chemically reactive substances that contain oxygen, such as superoxide anion radical (O_2_^•−^), hydrogen peroxide (H_2_O_2_), hydroxyl radical (OH^−^), ozone (O_3_), and singlet oxygen (^1^O_2_) (Apel and Hirt, 2004). O_2_^•−^ can be produced by NADPH oxidase (NOXs) or by the reduction in O_2_ by complexes I and III of the mitochondrial electron transport chain (ETC) (Murphy, 2009). Nicotinamide adenine dinucleotide phosphate (NADPH) produced through the pentose phosphate pathway is an important redox cofactor of the NOXs, which produce ROS (Sies and Jones, 2020). ROS produced by NADPH oxidase plays an important role in the plant’s participation in environmental response (Kawarazaki et al., 2013). Mitochondrial complexes III (CIII) and CI are necessary for ROS production (Fernández-Agüera et al., 2015). Studies have shown that Na can act as a second messenger to regulate the production of ROS by regulating the fluidity of the mitochondrial inner membrane (Hernansanz-Agustín et al., 2020). Excessive accumulation of ROS can be toxic, causing damage to carbohydrates, proteins, membranes, and genetic materials within plant cells. It inhibits normal plant growth, and development, and can even lead to plant death (Hossain et al., 2015; Jain and Gould, 2015). ROS production primarily occurs in chloroplasts and mitochondria through electron transport. Higher plants also produce ROS during metabolic processes such as fatty acid β-oxidation. Cold stress reduces the photosynthetic rate and affects photosynthetic electron transport, which affects ROS production (Bai et al., 2022). Living organisms position two antioxidant systems: enzymatic antioxidant systems and non-enzymatic antioxidant systems. Enzymatic antioxidant systems include peroxidase (POD), superoxide dismutase (SOD), catalase (CAT), ascorbate peroxidase (APX), and glutathione reductase (GR). Non-enzymatic antioxidant systems include flavonoids, phenols, carotenoids, vitamin C, vitamin E, glutathione (GSH), and ascorbic acid (ASA). Under normal temperature conditions, ROS production is kept at a low level in cell membranes, mitochondria, chloroplasts, and peroxisomes, with chloroplasts and mitochondria being the main sites of ROS production in plants (Neill et al., 2002). Studies on multiple plant species such as *Oryza sativa* (Guo et al., 2022), *Z. mays* (Zhao et al., 2022), *Manihot esculenta* Crantz (Liao et al., 2022), and *Capsicum annuum* (Wang et al., 2022) have shown that the activity of antioxidant enzymes is significantly enhanced under low temperature stress. In peanut seedlings, the activity of SOD and CAT shows a marked increase in response to cold stress, followed by an increase in the activity of POD before eventually decreasing. These antioxidant enzymes work together to maintain an appropriate level of active oxygen, which protects the plants against potential oxidative damage (Xue Yunyun et al., 2018). At low temperatures, full-length OsCYP20-2 in chloroplasts can improve the activity of superoxide dismutase OsFSD2, enhance the ability of cells to remove reactive oxygen species, and improve the cold tolerance of *O. sativa* (Ge et al., 2020a). In recent years, proteomic analysis of *B. napus* has shown that the molecular mechanism of the plant to enhance its tolerance to cold depends on the removal of ROS (Mi et al., 2021). It was reported that the activity of leaf antioxidant enzymes in plants under cold stress initially increased and then decreased over a certain period (Hongtao et al., 2017). Abscisic acid (ABA), an important plant hormone and growth regulator, has been found to improve cold tolerance by increasing the activity of antioxidant enzymes (Khorshidi and Nojavan, 2006). In the case of *Triticum aestivum*, treatment with ABA increases the activities of TaSOD, TaAPX, TaCAT, TaGR, TaDHAR, and TaMDHAR to enhance cold resistance (Yu et al., 2020). These findings highlight the importance of the redox system in plant response to low temperatures and the delicate balance between ROS production and scavenging for plant survival and adaptation to cold stress.

## 4 Molecular regulatory mechanism of plants’ response to low-temperature stress

### 4.1 Relations between plant hormones and cold resistance

Plant hormones are trace organic compounds produced by plants that play a crucial role in the regulation of their growth, development, and response to stress. These hormones include indole acetic acid (IAA), gibberellic acid (GA), cytokinin (CK), ABA, ethylene (ET), brassinosteroids (BR), JA, salicylic acid (SA), and strigolactones (SL). SA has been found to play a significant regulatory role in combating cold stress. A medium concentration of SA was applied to watermelon plants to induce cold tolerance by influencing the expression of the *CBF* and *COR* genes, improving the activity of antioxidant enzymes, and improving cold tolerance (Cheng et al., 2016). Combining SA and trisodium phosphate (TSP) improved fatty acid desaturation efficiency, improved water retention in *C. annuum*, and reduced membrane damage at low temperatures (Ge et al., 2020b). In *Z. mays*, *O. sativa*, and *C. sativus* leaves, treatment with 0.5 mM SA reduced cold-stress-induced electrolyte leakage and increased the activities of glutathione reductase and guaiacol peroxidase (G-POD) (Kang and Saltveit, 2002). ABA, which typically accumulates under cold stress, has been found to correlate with cold tolerance positively. The ABA content in cultivars with strong cold tolerance was significantly higher than in cold-sensitive cultivars (Huang et al., 2017; Agurla et al., 2018). The combination of low temperature and ABA induced the expression of *CBF/DREB1* in *Vitis vinifera* seedlings and increased cold tolerance in dormant buds when ABA was applied to the leaves (Rubio et al., 2019). ABA enhances cold tolerance by increasing the activity of antioxidant enzymes and regulating stomatal opening and closing, thereby reducing CO_2_ fixation and inhibiting the accumulation of reactive oxygen species (Lim et al., 2015). ABA and GA are the main endogenous regulatory factors, and ABA and GA have antagonistic effects on seed germination (Miransari and Smith, 2014). ABA induces dormancy, and GA regulates dormancy and germination. Cold stress during seed development enhances ABA accumulation and decreases GA content (Kendall et al., 2011; He et al., 2014). Low temperature regulates seed germination by mediating ABA and GA metabolism and signaling, and the two hormones antagonistically promote seed coat and endosperm rupture (MacGregor et al., 2015). As a plant growth regulator (PGR) that releases ethylene, ethephon can delay the flowering of many plants, greatly reducing the chance of freezing damage (Pahwa and Ghai, 2015). Ethephon can delay flowering and prolong the dormant period of flower buds by increasing cooling and improving the cold tolerance of flower buds (Durner and Gianfagna, 1991). The study showed that the appropriate application of ethylene prolongs the cooling accumulation period of peach buds, improves cold resistance, and delays flowering (Liu et al., 2021b). Low-temperature treatment induced the release of ethylene and the expression of *MdERF1B* (encoding an ethylene signal activator) in *M. domestica* seedlings. Overexpression of *MdERF1B* significantly enhances the cold tolerance of *M. domestica* and *A. thaliana* seedlings (Wang et al., 2021b). Ethylene can positively regulate the cold tolerance of *Poncirus trifoliata* (Zhang et al., 2022b). A-type response regulator *ZmRR1* in the CK signal transduction pathway is involved in the regulation of *Z. mays* cold tolerance, with natural variation in *ZmRR1* leading to differences in cold tolerance among *Z. mays* inbred lines (Zeng et al., 2021). JA and its derivatives, collectively known as jasmonate (Jas), are important molecules in regulating plant responses to biotic and abiotic stresses (Ruan et al., 2019). JA positively regulates leaves senescence, *CBF* expression, and downstream cold response genes, increasing cold tolerance in *O. sativa* (Du et al., 2013; Hu et al., 2017). A B-box protein (BBX) MdBBX37 was identified as a positive regulator of *M. domestica* cold stress resistance mediated by JA, shedding light on its function in jasmonate-mediated cold resistance (An et al., 2021). MeJA treatment increases cold tolerance, activates genes related to JA synthesis, increases endogenous JA content, and significantly increases antioxidant-related substance content in *C. annuum*, alleviating cold stress damage (Seo et al., 2020). BR regulates plant tolerance to low-temperature stress. It enhances cold tolerance by accumulating dephosphorylated forms of BZR1 and BES1, induces the expression of *CBF1* and *CBF2*, and positively regulates the cold tolerance of *A. thaliana. BZR1* acts upstream of *CBF1* and *CBF2*, directly regulating the expression of cold-tolerance-related genes (Li et al., 2017b). The phenylpropane group, including lignin, flavonoids, anthocyanins, and phenylpropane esters, is implicated in the response to cold stress. Upregulation of phenylpropanoid genes in *A. thaliana* seeds under cold stress is associated with a higher concentration of anthocyanins in the seed coat. Low temperatures can lead to cortical differences in tetrazolium, and low seed coat permeability or proanthocyanidin concentration can hinder seed dormancy after exposure to low temperatures (MacGregor et al., 2015). The early response genes of SL were identified in *A. thaliana*, and the molecular mechanism of SL anthocyanin accumulation and its regulation of plant growth and development was elucidated (Wang et al., 2020a). SL promotes the expression of the *CBF* gene and improves the cold tolerance of *A. thaliana* by promoting anthocyanin synthesis (Wang et al., 2023b). In summary, plant hormones such as SA, ABA, ethylene, CK, JA, BR, and SL play significant roles in the regulation of plant cold tolerance. Understanding the interactions between these hormones and their signaling pathways is crucial for enhancing plant cold resistance and improving crop productivity (**Table 1**).

**Table 1:** Examples of the role of plant hormones in cold stress responses.

Phytohormone		References
**Salicylic acid (SA)**	SA induces plant cold tolerance, affects the expression of *CBF* and *COR* genes, and improves antioxidant enzyme activity.	(Cheng et al., 2016)
	The desaturation efficiency of fatty acids can be improved by combining SA and TSP, enhancing water retention and alleviating membrane structure damage.	(Ge et al., 2020b)
	Cold tolerance is induced by SA, which enhances GR and G-POD activity.	(Kang and Saltveit, 2002)
**Abscisic acid (ABA)**	Enhanced cold tolerance is achieved through synergistic induction of *CBF/DREB1* expression by ABA and low temperature.	(Rubio et al., 2019)
	ABA enhances antioxidant enzyme activity, induces stomatal closure, and inhibits the accumulation of reactive oxygen species.	(Lim et al., 2015)
**Gibberellic acid (GA)**	Seed germination is regulated in an antagonistic manner by ABA and GA, promoting seed coat and endosperm rupture.	(MacGregor et al., 2015)
**Ethylene (ET)**	ET aids in cold resistance by enhancing cooling and prolonging the dormancy period of flower buds.	(Durner and Gianfagna, 1991)
	In *Malus domestica* seedlings, *MdERF1B* upregulates the expression of the low-temperature response gene *MdCBF1*. Additionally, *MdCIbHLH1* enhances the binding and transcriptional activation of *MdERF1B* to the target gene promoter in CBF-dependent pathways.	(Wang et al., 2021b)
	*PtrERF9*, induced by ethylene and low temperature, targets the ACC synthase gene *PtrACS1* in trifoliate citrus. This regulation affects the downstream target gene Glutathione S-transferase *PtrGSTU17* and ROS homeostasis in plants.	(Zhang et al., 2022b)
**Cytokinin (CK)**	*ZmRR1* enhances *Zea may’s* cold tolerance by positively regulating the expression of cold response genes such as *ZmDREB1* and *CesA*.	(Zeng et al., 2021)
**Jasmonic acid (JA)**	JA positively regulates *CBF*, upregulates JA biosynthesis-related genes (including *AOC*, *AOS1*, *AOS2*, and *LOX2*), and promotes cell responses to cold stress through signaling genes (*COI1a* and *bHLH148*).	(Du et al., 2013; Hu et al., 2017)
	MdBBX37 directly binds to the *MdCBF1* and *MdCBF4* promoters to promote their transcription. It also interacts with *MdICE1* to enhance the transcriptional activity of *MdICE1* toward *MdCBF1*, thereby enhancing cold tolerance.	(An et al., 2021)
	MeJA induces the activation of JA synthesis genes, increasing the content of antioxidant metabolites.	(Seo et al., 2020)
**Brassinosteroid (BR)**	BR accumulation leads to BZR1 and BES1 dephosphorylation levels, inducing *CBF1* and *CBF2* upstream pathways to regulate cold tolerance gene expression.	(Li et al., 2017b)
**Strigolactone (SL)**	SL activates *BRC1* expression, upregulates *HB40* expression level, and increases ABA content in lateral buds. It also upregulates the expression of Anthocyanidin synthesis genes *DFR*, *ANS*, and *TT7* by activating *PAP1*, *PAP2*, *MYB113*, and *MYB114*, promoting the synthesis and accumulation of Anthocyanidin.	(Wang et al., 2020a)
	SL inhibits the expression of *WRKY41*, promotes the interaction between MAX2 and WRKY41, and degrades WRKY41 through the 26S proteasome pathway. This leads to increased expression of *CBFs* and downstream genes, enhancing cold tolerance in *A. thaliana*.	(Wang et al., 2023b)

### 4.2 Epigenetic mechanisms

Low-temperature stress has been shown to induce epigenetic changes in plants, such as DNA methylation, chromatin modification, histone modification, and microRNA (miRNA) regulation. These epigenetic modifications play a crucial role in plant response to cold stress. For example, miRNA-mediated gene expression has been shown to play a crucial role in the response of plants to cold stress (Megha et al., 2018). MiRNA, a small noncoding RNA, regulates physiological processes and can help alleviate the harmful effects of abiotic stress on plants. The overexpression of *miR397a* induced the expression of cold-regulated *CBF* genes and downstream *COR* genes, thus improving the cold tolerance of *A. thaliana* (Dong and Pei, 2014). Upregulation of *miR393* under abiotic stress inhibits plant growth during stress, while cold stress leads to downregulation of *miR2004* in *A. thaliana* (Sunkar and Zhu, 2004). Cold-responsive miRNAs in *Z. mays* can regulate the size of *Z. mays* leaves under cold stress by modulating developmental transcription factors and maintaining redox homeostasis in plant cells (Aydinoglu, 2020). In *A. thaliana*, the overexpression of *miR402* can promote seed germination and enhance plant cold tolerance (Tiwari et al., 2020). OsmiR156 can enhance cold tolerance by targeting OsSPL3 to regulate the expression level of related transcription factor genes (Zhou and Tang, 2019). In *O. sativa*, OsmiR319 increases transcription levels of *OsDREB1A* by targeting OsPCF6 and OsTCP21, thereby clearing reactive oxygen species to improve plant cold tolerance (Wang et al., 2014). In general, only a few miRNAs may participate in plant cold tolerance through the CBF-COR pathway, laying a theoretical foundation for our study of the CBF-COR-independent pathway.

In *A. thaliana*, the highly expressed osmotic response protein HOS15 interacts with HD2C and binds to the promoter of *COR* genes, thus epigenetically regulating cold tolerance (Lim et al., 2020). Studies on *O. sativa* have shown that exposure to low temperatures can induce changes in the chromosome structure of the *OsDREB1b* promoter and upstream region. This can lead to histone H3 acetylation in the promoter regulatory region, altering the chromatin structure and impacting the cold tolerance of *O. sativa* (Roy et al., 2014). DNA methylation plays a crucial role in silencing endogenous gene transposons and regulating gene expression (Zhang et al., 2006). Cold stress can enhance the activity of transposons by altering the level of methylation of stress-responsive genes, leading to the induction of related cold resistance genes and enhanced cold tolerance. Significant changes in DNA methylation status have been observed in *Solanum lycopersicum* after exposure to low temperatures. Cold stress downregulates the expression of the plant DNA demethylase *DML2*, resulting in increased methylation levels in the promoter region. This leads to gene silencing, decreased flavor quality, and reduced volatile production (Zhang et al., 2016). Histone H3 trimethylation at lysine 27 (H3K27me3), a common chromatin modification, is pivotal in plant growth and stress resistance. *V. vinifera* leaves treated at 4°C exhibited changes in H3K27me3 modification. Genes targeted by H3K27me3 showed significantly reduced expression compared to non-targeted genes, indicating the suppressive effect of this modification on gene transcription across the entire genome. The methylation of relevant genomic proteins was detected through chip-seq binding transcriptome analysis, providing insights into the regulatory mechanism of H3K27me3 in response to cold stress (Zhu et al., 2023). The vernalization pathway plays an important role in the regulation of *FLC*. The histone demethylases JMJ30 and JMJ32 regulate the vernalization rate of *A. thaliana* by activating *FLC* (Maruoka et al., 2022). Therefore, understanding the molecular mechanisms of cold tolerance in plants, including epigenetic modifications and gene function, is a significant challenge that requires further research.

## 5 Summary and outlook of low-temperature resistance in plants

Temperature is a crucial environmental factor that significantly affects the growth of plants. Low temperatures can harm plants during winter, cause delays in sowing, and limit crop distribution. Therefore, it is imperative to enhance the cold resistance of plants and develop cold-resistant varieties for sustainable agricultural production in winter. Grafting is an alternative method to traditional breeding that can improve plants’ tolerance to environmental stress (Venema et al., 2008). Studying genes related to plant frost resistance is particularly significant because it allows us to use genetic engineering to enhance the frost resistance of non-hardy plants, with evident potential applications. Furthermore, using tolerant rootstocks during plant cultivation has been found to enhance resistance to low temperatures (Lee et al., 2010). However, there is still a lack of a comprehensive and efficient evaluation system for assessing cold tolerance in plants. Additionally, the molecular mechanism underlying the regulation of crop low-temperature and cold resistance regulatory proteins remains unclear.

Enhancing plant cold tolerance depends on various factors, including genes related to cold resistance, external environmental factors, and physiological mechanisms of cold resistance. Enriching germplasm resources for cold tolerance can be achieved by selecting varieties with excellent agronomic traits and quality and crossing them with varieties with strong cold resistance (Labroo et al., 2021). The determination of WCS120 protein content can also serve as a standard for selecting cold-resistant varieties (Vítámvás et al., 2010). The DHN5 protein, which is structurally similar to the WCS120 protein in wheat, can also be used as a marker for assessing frost tolerance (FT) in barley (Kosová et al., 2008; Kosová et al., 2013). The positive regulatory effect of CTB2 on cold tolerance in *O. sativa* has been confirmed through transgenic technology, contributing to the cultivation of cold-tolerant *O. sativa* germplasm resources (Li et al., 2021b). By closely examining QTLs associated with cold tolerance in *O. sativa* using genome-wide association studies (GWAS), researchers have conducted genetic analyses of cold tolerance-related genes, developed polymorphic molecular markers, and bred new cold-resistant varieties under cold stress conditions (Li et al., 2021a). Moreover, modern biotechnological advancements, including CRISPR/Cas9 technology, metabolomics, genomics, and proteomics, have facilitated genome sequencing in different cold-resistant plant varieties, enabling deeper investigations into the mechanisms of plant cold resistance. TALENs and CRISPR/Cas systems have revolutionized biological research, and their application to crop plants allows for targeting specific traits to improve crop productivity and ensure food security (Sedeek et al., 2019). Additionally, studies have shown that stimulating seeds with ROS nanoparticles (NPs) can enhance seed germination speed under stress conditions and improve seedling resistance to stress. Strategies based on this nanobiostimulant could promote sustainable agriculture by reducing the use of pesticides (Yan et al., 2023). This knowledge will facilitate the development of robust and resilient plant varieties that can withstand cold temperatures, ultimately addressing the issue of plant cold damage (**Table 2**).

**Table 2:** Cold stress-related genes.

Gene name	Species	Gene function	References
***MYB15***	*Arabidopsis thaliana*	MYB15 protein can interact with ICE1 and bind to the Myb recognition sequence of the *CBF* gene promoter. Overexpression of *MYB15* leads to reduced expression of the *CBF* gene and regulates plant cold tolerance.	(Agarwal et al., 2006a)
***bHLH002***	*Oryza sativa*	OsMAPK3 interacts with OsbHLH002 protein and phosphorylates it to activate *OsTPP1* and enhance the cold tolerance of rice.	(Zhang et al., 2017)
***PUB25/26***	*Arabidopsis thaliana*	Cold-activated OST1 phosphorylates PUB25 and PUB26, promoting the degradation of MYB15 and enhancing plant cold resistance.	(Wang et al., 2019)
***OST1***	*Arabidopsis thaliana*	Cold-activated OST1 phosphorylates BTF3L, enhances the interaction with CBF protein, and promotes the expression of *COR* genes.	(Ding et al., 2018)
***ICE1***	*Arabidopsis thaliana*	Cold-activated OST1 phosphorylation of ICE1 enhances its stability and transcriptional activity modulates plant cold tolerance.	(Ding et al., 2015)
***MPK3/6***	*Arabidopsis thaliana*	MPK3 and MPK6 interact with and phosphorylate ICE1, negatively regulating the cold tolerance of *Arabidopsis.*	(Li et al., 2017a)
***B1L***	*Arabidopsis thaliana*	B1L enhances cold tolerance by enhancing *CBF* stability, thereby inducing *COR* gene expression.	(Chen et al., 2019)
***BZR1***	*Arabidopsis thaliana*	*BZR1* triggers plant responses to cold stress by directly regulating the expression of *CBF1/2* or *COR* genes.	(Li et al., 2017b)
***Trx-h2***	*Arabidopsis thaliana*	Low-temperature-mediated translocation of h2-type thioredoxin can induce CBF structural transformation and functional activation through REDOX changes, thus improving plant cold tolerance.	(Lee et al., 2021)
***WRKY41***	*Arabidopsis thaliana*	WRKY41 can bind to the W-box motif of the *CBF* promoter region and negatively regulate *CBF* gene expression and *Arabidopsis* cold tolerance.	(Wang et al., 2023b)
***HY5***	*Brassica napus*	Hypocotyl length was closely related to cold tolerance. *HY5* showed positive regulation on hypocotyl elongation.	(Jin et al., 2022)
***BBX29***	*Arabidopsis thaliana*	BBX29 can inhibit the expression of some cold response genes independent of the CBF pathway through specific binding to cis-acting elements such as G-box, thereby negatively regulating the cold tolerance of plants.	(Wang et al., 2023a)
***NAC104***	*Malus domestica*	MdNAC104 enhanced plant cold tolerance by promoting anthocyanin synthesis and expression of genes encoding antioxidant enzymes.	(Mei et al., 2023)
***WRKY2***	*Cynodon dactylon*	*CdWRKY2* promotes cold tolerance by mediating sucrose biosynthesis and the CBF signaling pathway.	(Huang et al., 2022)
***PIF3***	*Arabidopsis thaliana*	*PIF3* regulates plant cold tolerance by negatively regulating the expression of CBF pathway genes.	(Jiang et al., 2017)
***CBF2***	*Arabidopsis thaliana*	*MbCBF2* positively regulates cold tolerance by enhancing the activity of antioxidant enzymes and the expression of downstream-related genes.	(Li et al., 2022a)
***MPK1/2***	*Solanum lycopersicum*	SIMPK1/2-mediated phosphorylation of SIBBX17 enhances CBF-dependent cold tolerance.	(Song et al., 2023)
***CBF1***	*Citrus sinensis*	*CsCBF1* directly regulates the expression of arginine decarboxylase (ADC) and the synthesis of putrescine (Put) to coordinate cold resistance.	(Song et al., 2021)
***CBF2/4***	*Tectona grandis*	Genetic transformation experiments showed that *TgCBF2* and *TgCBF4* improved the cold resistance of *Arabidopsis* plants.	(Liu et al., 2023)
***BIN2***	*Hevea brasiliensis*	*HbBIN2* regulates plant cold tolerance by regulating *HbICE1* transcriptional activity and ROS homeostasis.	(Qiu et al., 2023)
***CIPK18***	*Vitis amurensis*	*VaCIPK18* overexpression positively regulates cold tolerance by reducing the production of reactive oxygen species.	(Yu et al., 2022)
***CIPK13***	*Capsicum annuum*	Overexpression of *CaCIPK13* improves cold tolerance by increasing anthocyanin content and active oxygen-scavenging enzyme activity.	(Ma et al., 2021)
***NAM3***	*Solanum lycopersicum*	*SINAM3* regulates plant cold tolerance by increasing ethylene synthesis.	(Dong et al., 2022)
***TCP1***	*Chrysanthemum morifolium Ramat*	*DgTCP1* enhances cold tolerance by promoting peroxidase gene expression and reducing ROS accumulation.	(Li et al., 2022b)
***WRKY40***	***Kandelia obovata***	*KoWRKY40* endows transgenic *Arabidopsis* with cold tolerance by regulating the ICE-CBF-COR signaling pathway.	(Fei et al., 2022)

## Author contributions

YW: Writing – original draft, Writing – review & editing. JW: Writing – original draft, Writing – review & editing. RS: Writing – review & editing. WZ: Writing – review & editing. RG: Writing – review & editing. K-MZ: Writing – review & editing. X-LT: Writing – review & editing.

## Glossary

**Table:**

ABI	ABA insensitive
ACC	1-aminocyclopropane-1-carboxylic acid
ACS	1-aminocyclopropane-1-carboxylic acid synthase
ANS	anthocyanidin synthase
AOC	allene oxide cyclase
AOS	allene oxide synthase
BBX	B-box
BES	BRI1-EMS-suppressor
BRC	branched
bHLH	basic helix–loop–helix
BZR	Brassinazole-resistant
CBF	C-repeat binding factor
COI	coronatine insensitive
COR	cold regulated
DFR	dihydro-flavonol 4-reductase
DREB	dehydration-responsive element binding protein
ERF	ethylene response factor
G-POD	guaicol peroxidase
GR	glutathione reductase
GSTU	glutathione S-transferase U
HB	homeobox
HOS	high expression of osmotically responsive gene
HY	hypocotyl
ICE	inducer of CBF expression
LOX	lipoxygenase
MAX	more axillary growth
MYB	V-MYB avian myeloblastosis viral oncogene homolog
PAP	production of anthocyanin pigment
PIF	phytochrome-interacting factors
PUB	plant U-box
ROS	reactive oxygen species
RPL	ribosomal protein L
RR	response regulator
TSP	trisodium phosphate
TT	transparent testa
ZAT	zinc finger of *Arabidopsis thaliana*
BIN	BR-insensitive
CIPK	calcineurin B-like protein-interacting protein kinase
NAC	nascent polypeptide-associated complex
TCP	TEOSINTE BRANCHED1/CYCLOIDEA/PROLIFERATING.
